# Progress in Medicine

**Published:** 1900-01-13

**Authors:** 


### PROGRESS IN MEDICINE.

### DISEASES OF THE HEART AND CIRCULATION.

(*Continued from page* 227.)

**Effects of Athletics on the Heart.**—Stengel[1] examined nine members of a football team immediately after playing, and found a systolic pulmonary murmur in three—probably due to dilatation of the conus arteriosus of the right ventricle. This murmur disappeared after a rest. Muscular exercise, sufficiently rapid to cause breathlessness, puts a strain on the right ventricle, which in the robust and "trained" individual is soon recovered from, but in the middle-aged may bring about lasting disease. The question of "second wind" is a question of the right ventricle. Athletics continually indulged in tend to bring about some hypertrophy of the heart.

Williams[2] has also made observations on this subject, based on examination of the hearts of 17 vigorous men before and after a cross-country run of 25 miles. Very slight dilatation of the left ventricle was found, by percussion, in all after a rest. One curious fact was that this violent muscular exercise was attended by a low arterial tension. In 11 of the men there was a true mitral regurgitant murmur, which lasted from a few minutes to half an hour, and the men were not permanently injured.

**Angina Pectoris.**—Samberger[3] presents statistics of angina pectoris from the Czech polyclinic at Prague. He finds that the disease is not rare in out-patient practice—in workers of the labouring class. It is not confined to wealthy persons who fare sumptuously every day and do no physical work. Most patients are over 40 years of age. Most writers contend that true angina pectoris generally ends sooner or later in sudden death, and that recovery is a rare exception. Samberger finds, on the contrary, that this statement holds good only of patients in whom angina is combined with aortic insufficiency. Most of his patients after treatment recovered sufficiently to again undertake laborious work, and a few were completely cured.

**Fatty Heart.**—According to Bureau[4] the mechanism of sudden death in cases of fatty heart differs in the simple cases and in those complicated with atheroma of the coronary arteries. In the latter condition death is apt to be caused by a rupture of the heart, or occur during an attack of angina pectoris. In uncomplicated fatty heart death is not directly attributable to the heart, but is due to syncope, and the causes of that accident may be various. In young and otherwise healthy persons the fatal syncope may be brought about by some such cause as a forcible movement, a cold bath, an injury. In the anæmic or cachectic with a fatty heart it may be induced by cerebral anæmia, occasioned by a sudden change from a horizontal to a vertical position.

**Pericardium.**—Barnard[5] has made experiments to determine the functions of the pericardium. He states that it bears the same mechanical relation to the heart as the leather case of a football to the inside case. When a large quantity of venous blood is driven into the right heart the pericardium prevents over-distension. It limits the capacity of the heart by about one-half. The most frequent and powerful cause of sudden engorgement of the right side of the heart with blood is the active working of the abdominal muscles in respiration during violent exercise, raising the blood to the heart from the splanchnic blood-vessels, the action of which is added to that of the negative pressure within the thorax produced by every inspiration. It is in such violent exercise that the pericardium prevents over-distension of the heart. The pericardium becomes softened when inflamed. It is, therefore, of the greatest importance, in view of the functions of this membrane, to keep a patient suffering from pericarditis at absolute rest, not only until the inflammation has subsided, but until the inflammatory materials have organised into sound fibrous tissue. The dyspnœa of pericarditis with effusion is in reality a violent working of the respiratory force pump, sucking the blood into the heart with each inspiration, and expelling it through the aorta with each expiration. It is a struggle of the skeletal muscles to maintain the circulation through the heart in spite of the strangling pressure of the pericardial effusion. The condition can be imitated experimentally by injecting warm oil into the pericardium. It is found that no effect is produced while the oil fills the space in the pericardium which is not filled by the heart, but directly the auricles were pressed upon dyspnœa occurred. Directly the oil was allowed to escape the dyspnœa ceased. These experiments suggest that dyspnœa occurring in pericarditis with effusion is an indication for releasing the effusion. Pericarditis with effusion is very difficult to diagnose from a dilated heart, but in the latter condition there will probably be tricuspid regurgitation and systolic pulsation in the veins of the neck.

**Chronic Mediastinitis.**—Fresh cases of the rare complaint, chronic mediastinitis, have been recorded by Whipham[6] and Bosanquet,[7] and the former has, in addition, summarised our existing knowledge of the disease. It is of more frequent occurrence in adults than in children, probably due to the fact that it is chronic, and that a fatal termination does not, as a rule, take place until after a lapse of years. The causation is very doubtful; rheumatism is recorded in but a very small proportion of cases, and tuberculosis is rare. The pathological sequence of events is probably as follows: (1) Chronic inflammation of all serous membranes; (2) stagnation occasioned by degeneration of the cardiac muscle, leading to ascites, because on account of the chronic peritonitis the peritoneal vessels offer a locus minoris resistentive; (3) the cirrhotic process in the liver is occasioned by the extension of inflammatory irritation from the capsule of the liver as well as by the chronic hyperæmia of the organ. The disease does not in the majority of cases take its origin in any inflammation primarily affecting the heart, though in some instances changes occur in the surrounding tissues which are sufficient to call forth such an increased action of its muscular walls as to lead eventually to hypertrophy. The pericardium is usually more or less affected by the inflammatory process, and its cavity in the majority of cases is partially or entirely obliterated by fibrous adhesions. The pulse is always feeble and rapid, in the majority of cases 100 or over per minute. Pulsus paradoxus has been often observed, though in only one-half of the cases showing this feature a constriction of the large vessels by fibrous bands existed. Albumen is not a constant constituent of the urine, but is often present in greater or less quantity, and is dependent on the state of the circulation rather than organic changes in the kidneys. Ascites is generally present, due to the chronic peritonitis, and the liver is enlarged owing to chronic venous congestion.

The diagnosis of the condition is often a matter of considerable difficulty, and the cases are often mistaken for cirrhosis of the liver. The disease may be suspected if the upper limbs or face and neck are early involved. Cyanosis of the lips and œdema of the thoracic walls, occurring exceptionally or quite early, are important points in the diagnosis of the disease. Treatment, either medical or surgical, is of little avail when the pathological condition is fully established.

[1] Stengel, Med. Rec., May 13, 1899. [2] Williams, Med. Rec., May 13 1899. [3] Samberger, Sbornik Klinicky, Vol. i., 1899. [4] Bureau, Thèse de Paris, 1898. [5] Barnard, Lancet, April 22, 1899. [6] Whipham, Lancet, April 1 and 8, 1899. [7] Bosanquet, Lancet, July 1, 1899.

